# Passing Events and Topics

**Published:** 1921-01-08

**Authors:** 


### Passing Events and Topics.

### Royal College of Surgeons of England.

Six Hunterian Lectures on "The Principles of Human Craniology," illustrated by specimens and preparations, will be delivered by Professor Arthur Keith, M.D., LL.D., F.R.S., F.R.C.S., Conservator of the Museum, in the Theatre of the College in Lincoln's Inn Fields, at 5 p.m., on Mondays, Wednesdays, and Fridays, beginning January 17, and ending Friday, January 28.

### Lectures on Hospital Administration.

A three months' course of lectures and demonstrations in hospital administration began on Tuesday, January 4, at the Western Fever Hospital, Seagrave Road, Fulham, and will be held on successive Fridays and Tuesdays at 5 p.m. Dr. R. M. Bruce, medical superintendent of the Western Hospital, is the lecturer, and the course is stated to be suitable for those reading for a diploma in public health. The fee is £3 3s., which should be paid before the course is attended, to the Clerk to the Metropolitan Asylums Board, Embankment, London, E.C.

### Memorial to Sir Victor Horsley.

The first list of subscriptions, paid and promised, towards the foundation of a lectureship or scholarship to commemorate the services of the late Sir Victor Horsley to science and to the Empire has been published. The Hon. Treasurers are Sir Frederick Mott, K.B.E., 25 Nottingham Place, W. 1, and Dr. H. H. Tooth, C.B., C.M.G., 34 Harley Street; the Hon. Secretaries are Sir W. Arbuthnot Lane, Bart., 21 Cavendish Square, W. 1, and Edward Domville, Esq., C.B.E., Symondsbury, Bridport. Subscriptions may be paid to any of the honorary officers, or to the Victor Horsley Memorial Account, London County Westminster and Parr's Bank, Hanover Square, W. 1.

### A Record Boxing Tournament.

Just over £4,000 was netted by a recent boxing tournament promoted at Brighton by that excellent friend of hospitals, Mr. Harry Preston, of the Royal York Hotel, who himself defrayed all expenses, amounting to over £500. The latter arrangement had also the satisfactory result of saving about £1,100 in entertainment tax. All the local hospitals had a share, including £300 to the Royal Sussex County Hospital, and £700 to the Royal Alexandra Hospital for Children.

### L.C.C. and the Army Council.

During the war over 65,000 sick and wounded soldiers passed through the Horton and Manor Mental Hospitals, the Ewell Colony, and the Maudslay Hospital. The Council has received the thanks of the Army Council "for the generous and patriotic spirit displayed in placing certain of the Council's institutions under their control at the disposal of the Army Council during the war, and for the valuable assistance rendered in the administration of these institutions as military hospitals."

### Poor-Law Relief.

A recent White Paper issued by the Ministry of Health states that on January 1, 1900, the total number of persons in receipt of Poor-Law relief in England and Wales was 576,418, or one in 65 of the population. Women and children formed 76 per cent. of the total.

### Successful Pound Day at Mansfield.

Three tons of turnips, five tons of potatoes, 157 lb. of currants and raisins, 172 lb. of tea, and 1,375 lb. of sugar were among the articles received from Pound Day on behalf of the Mansfield and District Hospital, the total value of all the articles received being estimated at £200. Mr. Limb, the Secretary of the hospital, is also Secretary of the local Hospital Saturday and Sunday Funds, and his experience in the organisation and collection of small contributions stood the hospital in good stead. The teaching staff and scholars of the various schools in the district were particularly helpful in aiding the success of the effort.

